# Supplementary material for: Determination of Market, Field Samples, and Dietary Risk Assessment of Chlorfenapyr and Tralopyril in 16 Crops
Source: Foods. 2022 Apr 26;11(9):1246. doi: 10.3390/foods11091246 (PMC9102846; doi:10.3390/foods11091246)
Supplement: Supplementary file 1 [file foods-11-01246-s001.zip › foods-1664666-supplementary.pdf]

Determination of Market, Field Samples, and Dietary Risk Assessment of Chlorfenapyr and Tralopyril in 16 Crops

Hong Li [1], Fengshou Sun [2], Shuai Hu [1], Qi Sun [2], Nan Zou [1,2], Beixing Li [1,2], Wei Mu [1,2] and Jin Lin [1,2,*]

[1] Shandong Provincial Key Laboratory for Biology of Vegetable Diseases and Insect Pests, College of Plant Protection, Shandong Agricultural University, Taian 271018, China; 2018110350@sdau.edu.cn (H.L.); H15564801860@163.com (S.H.); zounan1226@163.com (N.Z.); libeixing@126.com (B.L.); muwei@sdau.edu.cn (W.M.)

[2] Research Center of Pesticide Environmental Toxicology, Shandong Agricultural University, Taian 271018, China; sfsun1990@163.com (F.S.); sunqi05172022@163.com (Q.S.)

* Correspondence: linjin@sdau.edu.cn; Tel.: +86-0538-8242611

Table S1. Sampling sites of the monitoring market.

Sampling sites		
Eight large supermarkets	Five small supermarkets	Seven farm markets
Yabo RT-Mart	Tesco Lifestyle Supermarket	Provincial Farmer's Market
RT-Mart	Four Seasons Supermarket	Wuma Farmer's Wholesale Market
New Age Supermarket	Wide Supermarket	Aolai Peak Farmer's Market
Jiayue Supermarket	Lattice Supermarket	Yingsheng Farmer's Market
Yonghui Superstores	Rundu Supermarket	Nan Men Farmer's Market
From Best Supermarket	-	Zaohang Farmer's Wholesale Market
Ginza Supermarket	-	Baibayu Convenient Market
C 'mon Lotus Supermarket	-	-

Table S2. Field test sites, crop varieties, and test types.

Test address	Longitude and latitude	Abbreviation of test sites	Crop varieties	Test types
Dongningwei Township, Taizihe District, Liaoyang City, Liaoning Province	123.25° E, 41.48° N	Liaoning Province	Lvyuan	Terminal residue tests
Dongyang Town, Yuci District, Jinzhong City, Shanxi Province	112.43° E, 37.41° N	Shanxi Province	Xingshu 16-2	Terminal residue tests
Baishan Town, Changping District, Beijing	116.46° E/39.92° N	Beijing	Zhonggan21	Residual dissipation and terminal residue tests
Taishan District, Taian City, Shandong Province	118.44° E, 36.11° N	Shandong Province	Shenglv168	Terminal residue tests
Xinbang Town, Songjiang District, Shanghai	121.29° E/31.14° N	Shanghai	Cattle cabbage	Residual dissipation and terminal residue tests
Dayang Town, Hefei City, Anhui Province	117.17° E, 31.52° N	Anhui Province	Super dong feng	Terminal residue tests
Chunhua Town, Changsha City, Hunan Province	112.59° E, 28.12° N	Hunan Province	Zhonggan19	Residual dissipation and terminal residue tests
Duchang Town, Duchang County, Jiujiang City, Jiangxi Province	115.58° E, 29.43° N	Jiangxi Province	Hazeline	Terminal residue tests
Xixiangtang District, Nanning city, Guangxi Province	108.19° E, 22.48° N	Guangxi Province	Flat cabbage	Terminal residue tests
Xiema Town, Beibei District, Chongqing	106.16° E, 29.18° N	Chongqing	Zhonggan11	Terminal residue tests
Jinzhu Town, Huaxi District, Guiyang, Guizhou Province	106.42° E, 26.35° N	Guizhou Province	Oxheart cabbage	Terminal residue tests
Mingcheng Town, Gaoming District, Foshan, Guangdong Province	113.06° E, 23.02° N	Guangdong Province	Zhonggan 21	Residual dissipation and terminal residue tests

Table S3. Purification combination and dosage of PSA, C_{18}, and GCB in 5 typical substrates.

Types	Matrix	Purifier combination	Ratios of purification agents (mg)
Vegetable and fruit samples	Cabbage<br>Apple	PSA+C_{18}	10+40<br>20+30<br>40+10
Grain and oil samples	Wheat<br>Peanut	PSA+C_{18}	10+40<br>20+30<br>40+10
Tea samples	Tea	PSA+C_{18}+GCB	10+40+5<br>20+30+5<br>40+10+5

Table S4. The gradient elution program.

Time (min)	Flow rate (mL min^{-1})	Percentage of mobile phase (%)	
		0.05% formic acid water (mobile phase A)	Methanol (mobile phase B)
0	0.30	90	10
2.50	0.30	2	98
3.50	0.30	2	98
3.60	0.30	90	10
5.00	0.30	90	10

Table S5. Determination conditions of tralopyril by mass spectrometry.

Active ingredient	Retention time (min)	Detection of ion pairs (m/z)	CE (V)
Tralopyril	2.95	348.98/80.99*	32.93
		348.98/131	37.6

Note: "*"is quantitative ion transition.

Table S6. Determination conditions of chlorfenapyr by mass spectrometry.

Active ingredient	Retention time (min)	Detection of ion pairs (m/z)	CE (V)
Chlorfenapyr	6.2	59.1/27.1*	20
		59.1/31.1	5

Note: "*"is quantitative ion transition.

Table S7. Final residue of chlorfenapyr on cabbage in 12 regions with an application dose of 120 g ha^{-1}.

Test site	Application times	Application interval (d)	Harvest interval (d)	Residual quantity (mg kg^{-1})			
				Repeat 1	Repeat 2	Repeat 3	Average value
Liaoning	2	7	14	0.201	0.219	0.211	0.210
			21	0.0428	0.0501	0.0445	0.046
Shanxi	2	7	14	0.31	0.309	0.312	0.310
			21	0.0292	0.0305	0.0332	0.031
Beijing	2	7	0	1.05	1.13	1.01	1.063
			3	0.534	0.489	0.498	0.507
			7	0.266	0.221	0.257	0.248
			14	0.0658	0.0655	0.0709	0.067
			21	< 0.01	< 0.01	< 0.01	< 0.01
Shandong	2	7	14	0.0661	0.0673	0.0768	0.070
			21	< 0.01	< 0.01	< 0.01	< 0.01
Shanghai	2	7	0	0.623	0.607	0.611	0.614
			3	0.482	0.503	0.535	0.507
			7	0.0691	0.0632	0.0678	0.067
			14	0.0185	0.0143	0.0155	0.016
			21	< 0.01	< 0.01	< 0.01	< 0.01
Anhui	2	7	14	0.118	0.118	0.126	0.121
			21	0.0147	0.0113	0.0154	0.014
Hunan	2	7	0	0.722	0.686	0.743	0.717
			3	0.514	0.503	0.535	0.517
			7	0.0638	0.0581	0.0558	0.059
			14	< 0.01	< 0.01	< 0.01	< 0.01
			21	< 0.01	< 0.01	< 0.01	< 0.01
Jiangxi	2	7	14	0.163	0.253	0.223	0.213
			21	< 0.01	< 0.01	< 0.01	< 0.01
Guangxi	2	7	14	0.066	0.0975	0.102	0.089
			21	< 0.01	< 0.01	< 0.01	< 0.01
Chongqing	2	7	14	0.338	0.342	0.332	0.337
			21	< 0.01	< 0.01	< 0.01	< 0.01
Guizhou	2	7	14	0.0163	0.0129	0.0167	0.015
			21	< 0.01	< 0.01	< 0.01	< 0.01
Guangdong	2	7	0	2.51	2.52	2.55	2.527
			3	1.49	1.44	1.42	1.450
			7	0.931	0.916	0.935	0.927
			14	0.459	0.488	0.466	0.471
			21	0.146	0.142	0.144	0.144

Table S8. MRLs, market monitoring of residues, and STMR in related crops.

Registered crop	Food type	MRL (mg kg^{-1})	Market monitoring of residues (mg kg^{-1})	STMR (mg kg^{-1})
Tea	salt	20	0.021	—
Chinese Cabbage	light vegetables	2	0.112	—
Beans	light vegetables	—	0.048	—
Cabbage	light vegetables	1	0.104	0.105
Citrus	fruits	—	—	—
Cucumber	light vegetables	0.5	0.246	—
Ginger	soy sauce	—	0.111	—
Hairy gourd	light vegetables	—	—	—
Cabbage mustard	dark vegetables	0.1	0.462	—
Leek	dark vegetables	—	0.262	—
Pear	fruits	—	—	—
Apple	fruits	—	—	—
Eggplant	light vegetables	1	0.192	—
Welsh onion	light vegetables	—	—	—
Pak choi	dark vegetables	10	0.281	—
Cowpea	light vegetables	—	0.504	—
Mulberry	fruits	2	—	—

Note:"*" means chlorfenapyr has not been registered on the crop; "-" means that the item has no data.

Total ion chromatogrm(TIC):

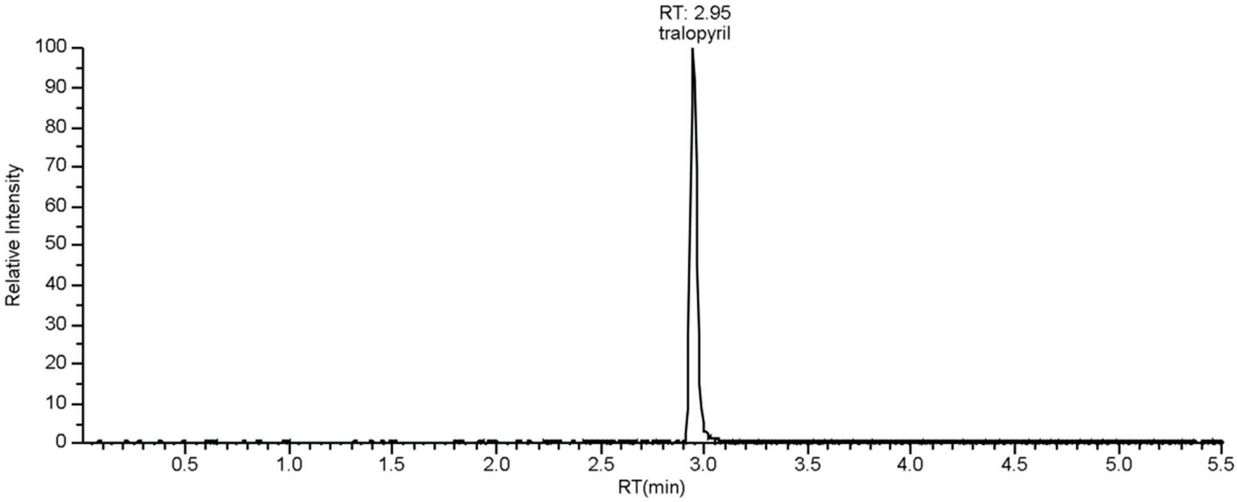

Qual Ion : 80.988 mz

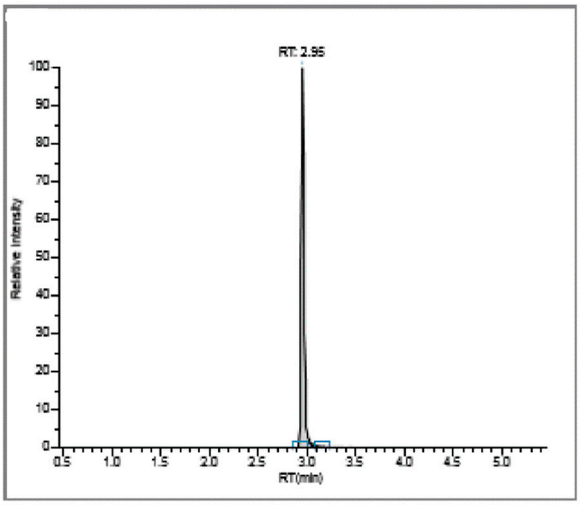

m/z 131.000

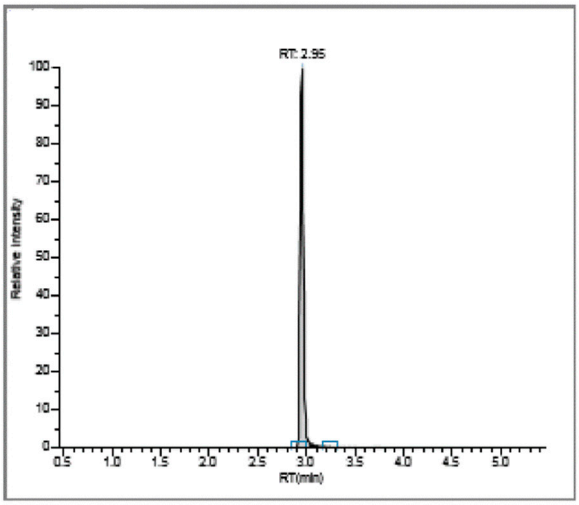

Figure S1. UHPLC-MS/MS chromatogram of tralopyril at a spiked level of 0.1 mg L^{-1}.

Total ion chromatogrm(TIC):

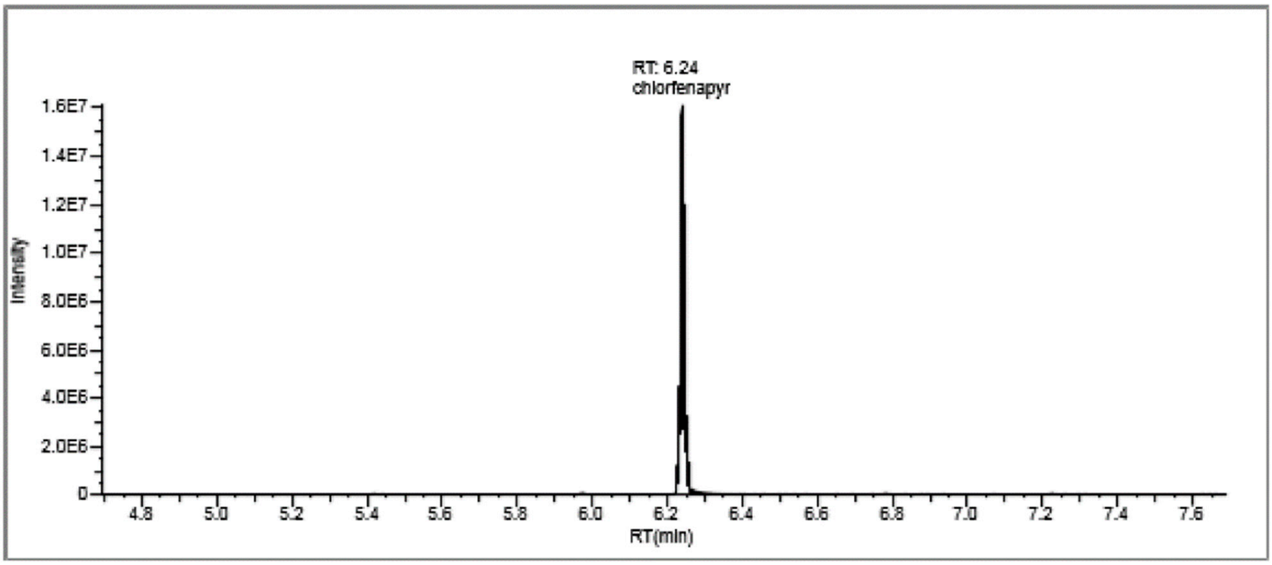

Qual Ion : 27.100 mz

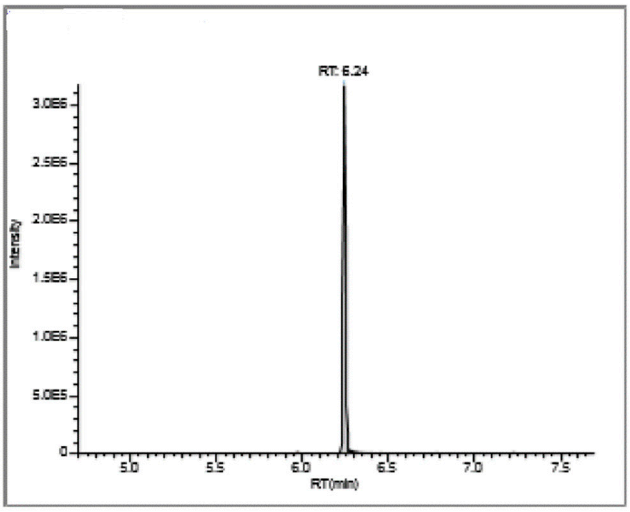

Qual Ion : 31.100 mz

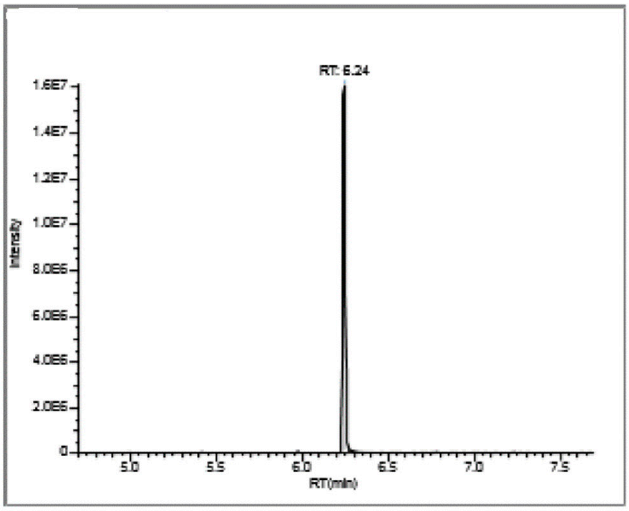

Figure S2. GC-MS/MS chromatogram of chlorfenapyr at a spiked level of 0.1 mg L^{-1}.
